# Iodine Intake Increases IP-10 Expression in the Serum and Thyroids of Rats with Experimental Autoimmune Thyroiditis

**DOI:** 10.1155/2014/581069

**Published:** 2014-02-25

**Authors:** Si-lu Cui, Jun Yu, Liu Shoujun

**Affiliations:** ^1^Institute for Iodine Deficiency Disorders Control, Center for Endemic Disease Control, Chinese Center for Disease Control and Prevention, Key Lab of Etiology and Epidemiology, Education Bureau of Heilongjiang Province & Ministry of Health, Harbin 150081, China; ^2^Institute for Kashin-Beck Disease Control, Center for Endemic Disease Control, Chinese Center for Disease Control and Prevention, Harbin Medical University, 157 Bao Jian Road, Harbin 150081, China

## Abstract

Here, we sought to establish an experimental autoimmune thyroiditis rat model induced by bovine thyroglobulin (bTg) injection and to investigate pathological changes and variations in serum interferon-**γ**-inducible protein of 10 kDa (IP-10) in thyroid tissue following iodine treatment. Four-week-old female Lewis rats (*n* = 135) were randomly divided into normal (NC), thyroglobulin (TG), HI, HI+TG, HII, and HII+TG groups; rats in the NC and TG groups drank only distilled water (iodine concentration: 10 **μ**g/L), rats in the HI and HI+TG groups were given water containing 25.7 mg/L iodine, and rats in the HII and HII+TG groups were given water containing 423.3 mg/L iodine. Rats in the TG, HI+TG, and HII+TG groups were immunized with 0.1 mL bTg (8 mg/mL) in incomplete Freund's adjuvant once every 2 weeks for 6 weeks. Compared with the NC group, the TG, HI+TG, and HII+TG groups exhibited higher iodine intake and increased thyroid weights with increasing iodine doses (*P* < 0.05). The high iodine intake in the TG group was associated with increased CD4^+^ T cells and serum IP-10. Thus, high iodine consumption aggravated the inflammatory reaction in the thyroid and mild high iodine consumption increased serum IP-10 levels after induction with bTg.

## 1. Introduction

Iodine is an essential component of thyroid hormone; low or high intake of iodine may lead to thyroid disease. Although iodine is an important exogenous predisposing factor of autoimmune thyroid disease (AITD) [1, 2], the pathogenic mechanisms through which iodine affects AITD are unclear. Experimental autoimmune thyroiditis (EAT) animal models have been used to study human Hashimoto's thyroiditis (HT) [3], an AITD that occurs in patients worldwide. Hakaru Hashimoto, the researcher who first characterized HT, summarized the four histological characteristics of this disease as the formation of lymphoid follicles, marked changes in thyroid epithelial cells, extensive formation of new connective tissue, and diffuse round cell infiltration [4].

Chemokines play a role in the selective recruitment of activated lymphocytes. Additionally, chemokine expression has been shown to be correlated with the presence of tissue inflammatory infiltrates [5]. Inducible protein of 10 kDa (IP-10), a chemokine of the CXC subfamily, is induced by interferon (IFN)-*γ* during inflammation and displays potent lymphocyte chemotactic activity.

In this study, we sought to investigate IP-10 expression in EAT rats following treatment with different doses of iodine and to investigate pathological changes in thyroid tissue, iodine intake, and expression of the serum chemokine IP-10.

## 2. Methods

### 2.1. Animals

Four-week-old female Lewis rats (specific pathogen-free), weighing approximately 80 g each, were purchased from Beijing Vital River Laboratory Animal Technology Company (Beijing, China). The rats (*n* = 135) were randomly divided into six groups according to weight: the normal (NC, distilled water, *n* = 20), thyroglobulin (TG, distilled water, *n* = 25), HI (iodine concentration in drinking water: 25.7 mg/L, *n* = 20, mildly high iodine), HI+TG (*n* = 25), HII (iodine concentration in drinking water: 423.3 mg/L, *n* = 20, severely high iodine), and HII+TG (*n* = 25) groups. Rats in the TG, HI+TG, and HII+TG groups were immunized by subcutaneous injection of 0.1 mL of bovine TG (bTg, 8 mg/mL) in incomplete Freund's adjuvant (Sigma Co., St. Louis, MO, USA) once every 2 weeks for a total of 6 weeks.

### 2.2. Level of Iodine Intake

Urine was collected from rats 2 weeks prior to euthanasia and stored at −20°C. A special cage was used to gather the urine of the rats over a 24-hour period. Urine iodine levels were determined by arsenic-cerium catalytic spectrophotometry [6]. Results are expressed directly as *μ*g/L urine. All rats were sacrificed at 15 weeks after immunization. The experimental scheme was optimized and the animals were treated humanely by improving the experiment method and adjusting the observation index of the experiment to ensure the implementation of animal welfare measures. The animal experiments were conducted per the ethical guidelines of the Medical Ethics Review Committee of Harbin Medical University.

### 2.3. Pathological Changes in Thyroid Tissue and Relative Thyroid Weights

After euthanization, the weights of the body and thyroid were measured, and the relative thyroid weight was expressed as the thyroid wet weight (mg)/rat body mass (g). The thyroid tissue was fixed with 4% paraformaldehyde and embedded in paraffin. The tissues were then sectioned into 0.4 *μ*m thick sections. All diagnoses were based on standard and widely accepted criteria [7].

The pathological features of the rat thyroids were classified into four grades, according to the grading standard of Roubaty et al. [8]: “−” indicated normal physiology; “+” indicated slight disruption of thyroid follicles, with several lymphocytes attacking two or more thyroid follicles; “++” indicated moderate destruction of thyroid follicles, with focal derangement of follicles occurring in 10–40% of thyroid sections; and “+++” indicated severe disruption of thyroid physiology, with diffuse lymphocytic infiltration occurring in >40% of thyroid sections.

### 2.4. Immunohistochemical Staining

The thyroid tissue was immersion-fixed in 4% paraformaldehyde at room temperature and then processed into paraffin sections by using a routine procedure. Endogenous peroxidase was blocked with 0.3% H_2_O_2_ in PBS [9]. No antigen-retrieval methods were used. Sectioned thyroid glands were subjected to immunohistochemical staining for CD4^+^ T cells using a 1 : 50 dilution of a rabbit polyclonal antibody (Abbiotec Co., USA). Blocking serum, secondary antibodies, and ABC reagents were obtained from Boster Co. (China). Photomicrographs were taken using a microscope (Olympus, Japan). CD4^+^ T cells were observed as yellow or brown staining products, and appropriate negative controls were used throughout.

### 2.5. Antibody Capture Enzyme-Linked Immunosorbent Assay (ELISA)

Blood sera were collected and stored at −80°C. Serum IP-10 levels were determined with sandwich ELISA, with a sensitivity ranging from 16 to 1000 pg/mL (Abnova Co., USA). ELISAs were performed according to the manufacturer's instructions. The absorbance was determined at 450 nm, and quantification was performed using standard curves.

### 2.6. Real-Time Fluorescent Quantitative Polymerase Chain Reaction (PCR)

Total RNA from the thyroid was extracted using TRIzol reagent (Life Technologies, NY, USA). Real-time PCR was conducted using SYBR Premix Ex Taq (Perfect Real-time PCR, Takara, Japan). The primer sequences were as follows: IP-10, forward 5′-CAAGGCTTCCCAATTCTC-3′ and reverse 5′-ACCTGGACTGCATTTGA-3′; GAPDH, forward 5′-GGCACAGTCAAGGCTGAATG-3′ and reverse 5′-ATGGTGGTGAACGCCAGTA-3′. The IP-10 primers yielded an amplification product of 202 bp, whereas the GAPDH primers yielded an amplification product of 143 bp. The PCR protocol was conducted as described by the manufacturer of the SYBR Premix Kit, with annealing temperatures of 57°C and 64°C for the IP-10 and GAPDH primers, respectively.

### 2.7. Statistical Analysis

Relative thyroid weight, serum IP-10 level, and urine iodine level have been reported in terms of the means ± standard deviation (SD). The data obtained were analyzed using an analysis of variance (ANOVA) with SPSS 17.0 software. The rank-sum test was used to analyze pathological changes in the thyroid.

## 3. Results

### 3.1. Urinary Iodine Level and Relative Thyroid Weight following Iodine Treatment in EAT Rats

**Figure 1:**
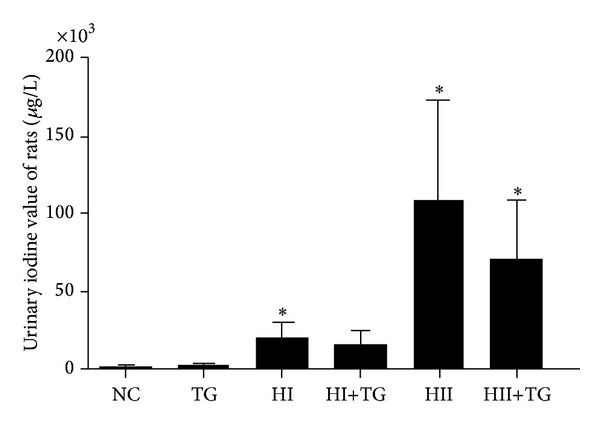
The bars in this figure show different urinary iodine values in each group of rats. (*F* = 44.428, *P* < 0.05). Compared with the NC group, each group showed an increase in the urinary iodine value with increase in the iodine intake (*P < 0.05 compared with the NC group).

Compared with the NC group, the HI, HII, and HII+TG groups exhibited increased urinary iodine levels (*P* < 0.05), and the urinary iodine levels increased as iodine intake increased (Table 1 and Figure 1).

There were no significant differences in body weight or thyroid weight among experimental groups. However, compared with the NC group, all experimental groups exhibited increased relative thyroid weights (*P* < 0.05, Table 2).

### 3.2. Pathological Features of Rat Thyroids

**Figure 2:**
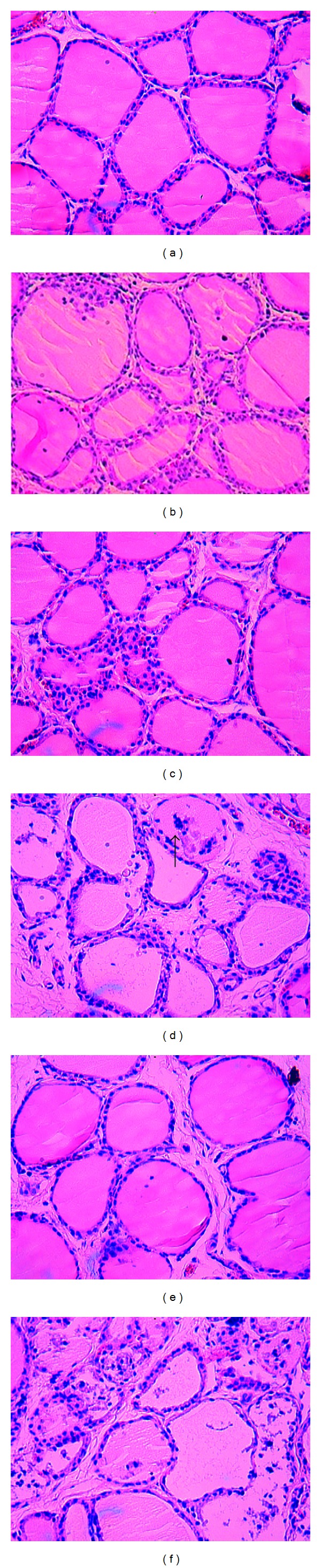
Histological appearance of rat thyroid tissue. (a) Section of thyroid tissue from the normal group, showing absence of lymphocyte cell infiltration (−). (b) Section of thyroid tissue from the TG group (+). (c) Section of thyroid tissue from the HI group (+). (d) Many mononuclear cells infiltrating the thyroid tissue from the HI+TG group (++). *Arrows* point to lymphocyte cell infiltration. (e) Section of thyroid tissue from the HII group, showing diffuse thyroidization (++). (f) Section of thyroid tissue from the HII+TG group (+++). Original magnification: HE, 200x.

Thyroid follicles in rats in the NC group were similar in size, development, and maturation and exhibited a lobular pattern (Figure 2). However, in the TG and HI groups, the follicles showed focal derangement and the presence of lymphocytes. The follicles of rats in the HI+TG and HII groups exhibited moderate pathological changes, with lymphocytes and fibrous tissue proliferation present among the follicles. Rats in the HII+TG and HII groups showed diffuse follicle destruction and diffuse lymphocytic infiltration. Additionally, the follicles differed in size (Table 3).

### 3.3. Expression of CD4^+^ T Lymphocytes in Rat Thyroid Tissue

**Figure 3:**
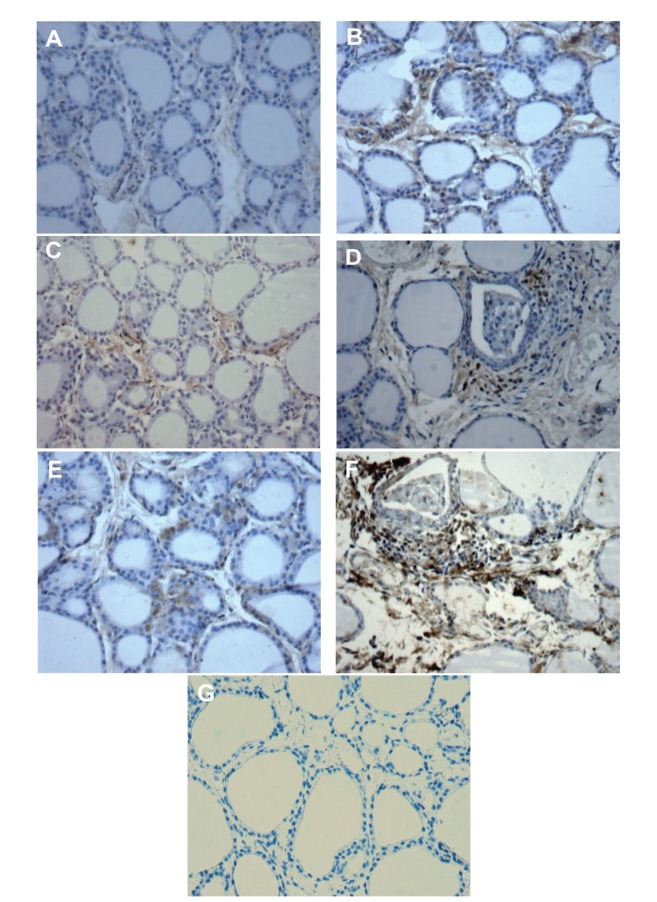
CD4^+^ T cell expression and localization in thyroid tissues from EAT rats. (A) Section of thyroid tissue from the normal group, showing absence of staining for CD4^+^ T cells. (B) Section of thyroid tissue from the TG group, showing immunostaining for CD4^+^ T cells in many mononuclear cells (yellowish-brown). (C) Section of thyroid tissue from the HI group. (D) Immunostaining for CD4^+^ T cells in many mononuclear infiltrating cells in the thyroid tissue from the HI+TG group. (E) Section of thyroid tissue from the HII group. (F) High immunoreactivity for CD4^+^ T cells in thyroid tissues from the HI group. (G) Section of thyroid tissue with negative control staining. Original magnification: 200x.

Immunohistochemical staining was used to identify the expression of CD4^+^ T lymphocytes in rat thyroid tissues. No staining of CD4^+^ T lymphocytes was observed in thyroid tissues from rats in the NC group, and few scattered infiltrating inflammatory cells were observed in the TG and HI groups. However, the HII+TG and HII groups showed diffuse CD4^+^ T lymphocytic infiltration (Figure 3).

### 3.4. Expression of IP-10 Protein in Rat Serum and IP-10 mRNA in Thyroid Tissues

**Figure 4:**
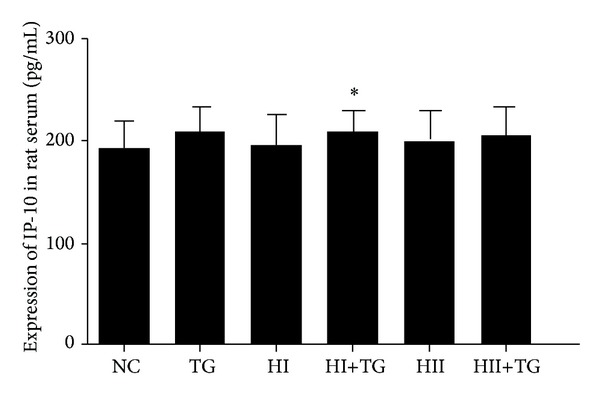
Bars in this figure show differential expression of IP-10 in the serum of rats in each group. Expression of serum IP-10 was measured using ELISA. Serum IP-10 levels in the HI+TG group were significantly higher than those in the NC group.

**Figure 5:**
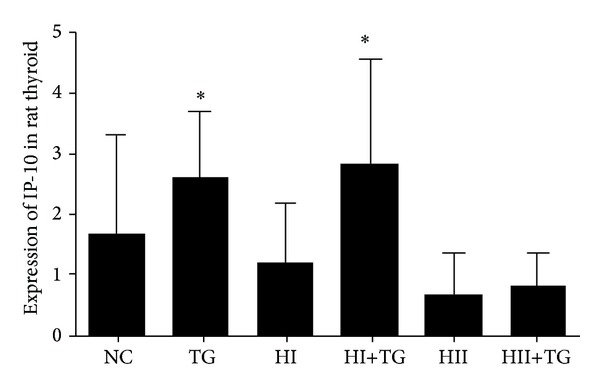
Bars in this figure show differential expression of IP-10 mRNA in the rat thyroid in each group. Expression of IP-10 mRNA in the thyroid tissues of rats in the HI and HI+TG groups was significantly higher than that in the NC group.

Compared with the NC group, the HI+TG group exhibited increased expression of serum IP-10. Moreover, IP-10 levels rose as iodine intake increased and rats in the TG, HI+TG, and HII+TG groups exhibited higher levels of serum IP-10 than corresponding control rats not given bTg injections, but the differences did not reach statistical significance (Table 4 and Figure 4). In the thyroid gland, rats in the TG and HI groups showed higher expression of IP-10 mRNA than the NC group, whereas the HII+TG group showed lower IP-10 expression (Table 5 and Figure 5).

## 4. Discussion

AITD is a multifactorial disease whose etiology involves both genetic susceptibility and environmental factors [10]. In this study, we found that high iodine intake aggravated the inflammatory reaction in the thyroid gland and increased serum IP-10 levels in Lewis rats after induction of thyroiditis by bTg injection. Therefore, we selected Lewis rats to establish our EAT rat model. Because urinary iodine accounts for 80–90% of the body's total iodine excretion, urinary iodine can be used as an indicator of iodine intake. Excessive iodine intake may lead to hypothyroidism and AITD [11]. In this study, the urine iodine concentrations of rats from the high iodine groups were higher than those of rats in the NC group and increased as the concentration of iodine in the rats' drinking water increased. Thus, consistent with a previous study, we found that high iodine intake aggravated the inflammatory reaction in thyroid glands of Lewis rats after induction of EAT by bTg injection and iodine consumption.

The EAT animal model can be considered representative of human HT. For establishment of this model, we used both excessive iodine intake and TG injection. We observed different levels of lymphocyte infiltration and thyroid follicle destruction following consumption of various concentrations of iodine, indicating successful establishment of the EAT rat model. Rats exhibited severe pathological changes in the thyroid gland as the concentration of iodine increased and with successive TG injections. Moreover, rats in the HII+TG group subjected to high iodine intake and TG injections showed a higher prevalence of EAT symptoms and more severe pathological changes than rats in the NC group. Compared with rats given only excessive iodine or TG injections, the use of both treatments induced more severe thyroiditis. Therefore, our study demonstrated that optimal establishment of the EAT rat model was achieved when rats were given drinking water with a high (423.3 mg/L) iodine content and simultaneously immunized with bTg (8 mg/mL).

T lymphocytes play an important role in human HT and cellular and humoral immunity in EAT rats [12]. The T cell subset that has attracted the most attention in this context is CD4^+^ T cells [13]. EAT rats mainly have CD4^+^ T lymphocytes, which present features of activated type-1 T helper cells (Th1 cells). CD4^+^ T lymphocytes secrete cytokines that stimulate cellular phagocytosis. Thus, for further verification of pathological changes in the thyroid gland, we used immunohistochemical staining to identify the inflammatory cells within the thyroid tissue. Microscopic examination of thyroid gland sections showed no evidence of CD4^+^ T lymphocytes in NC rats; however, CD4^+^ T lymphocytes were observed in model rats, indicating lymphocytic infiltration in thyroid sections. Additional studies are required to further investigate this phenomenon.

Chemokines are a family of small, structurally related molecules that regulate cell trafficking in various types of leukocytes through interactions with seven-transmembrane G protein-coupled receptors. Thyroid follicular cells (TFCs) produce CC and CXC chemokines, which promote the initiation and maintenance of the inflammatory process, resulting in AITD. A high level of IP-10 in peripheral fluids is therefore a marker of the host immune response, especially T helper (Th)1 T-cells [14]. Excessive levels of iodine intake have been reported to increase the incidence and prevalence of AITD in humans. The association of high IP-10 levels with iodine intake in the thyroid gland in EAT model rats is readily explained by the presence of serum IP-10 and IP-10 mRNA in the thyroid. Antonelli et al. [15] reported that high levels of circulating CXC chemokine ligand 10 are associated with chronic autoimmune thyroiditis, and our results corroborated these conclusions.

## 5. Conclusion

In conclusion, our results indicate that excessive iodine intake aggravated inflammatory infiltration and increased IP-10 expression in the serum of EAT rats. Additionally, iodine intake resulted in increased CD4^+^ T cells in the thyroid, demonstrating pathological changes in the target tissue.

## Figures and Tables

**Table 1:** Urinary iodine values in test rats (mean ± SD).

Groups	*n*	Urinary iodine value (*μ*g/L)
NC	19	569.66 ± 440.28
TG	23	943.05 ± 553.87
HI	18	20778.17 ± 8619.18*
HI+TG	24	15180.41 ± 8054.43
HII	19	111065.61 ± 61257.53*
HII+TG	22	70767.81 ± 38796.19*

*P < 0.05 compared with the NC group.

**Table 2:** Body weights and thyroid weights in model rats (mean ± SD).

Group	Body weight (g)	Weight of thyroid (mg)	Relative weight of thyroid (mg/g)
NC	245.05 ± 22.66	20.8 ± 2.7	0.08525
TG	251.92 ± 12.84	24.0 ± 2.5	0.09522*
HI	248.74 ± 14.59	23.0 ± 2.9	0.09285*
HI+TG	256.28 ± 20.59	24.9 ± 2.9	0.09748*
HII	244.37 ± 22.88	23.5 ± 2.8	0.09655*
HII+TG	245.20 ± 12.87	23.4 ± 2.7	0.09533*

*P < 0.05 compared with the NC group. The relative thyroid weight has been expressed as thyroid wet weight (mg)/rat body mass (g).

**Table 3:** Grades of thyroid pathological features in EAT rats.

Group/grade	*N*	−	+	++	+++
NC	9	8	1	0	0
TG^a^	19	8	8	3	0
HI^ab^	19	3	8	6	2
HI+TG^ab^	21	5	4	12	0
HII^a^	18	4	7	7	0
HII+TG*	20	1	0	10	9

^a^ *P* < 0.05 compared with the NC group; ^b^ *P* < 0.05 compared with the TG group; *P < 0.05 compared with each group.

**Table 4:** Expression of IP-10 in rat serum (mean ± SD).

Group	*n*	IP-10 (pg/mL)
NC	18	192.49 ± 25.7
TG	20	208.88 ± 24.6
HI	16	194.97 ± 29.9
HI+TG	21	209.36 ± 16.3*
HII	19	197.52 ± 30.3
HII+TG	18	203.91 ± 28.3

*P < 0.05 compared with the NC group.

**Table 5:** Expression of IP-10 mRNA in the rat thyroid (mean ± SD).

Group	*n*	IP-10 mRNA
NC	9	1.50 ± 1.64
TG	19	2.54 ± 1.00*
HI	19	1.07 ± 1.00
HI+TG	21	2.80 ± 1.73*
HII	18	0.64 ± 0.64
HII+TG	20	0.81 ± 0.49

*P < 0.05 compared with the NC group.
